# Do sheep (*Ovis aries*) discriminate human emotional odors?

**DOI:** 10.1007/s10071-024-01895-1

**Published:** 2024-07-26

**Authors:** Izïa Larrigaldie, Fabrice Damon, Solène Mousqué, Bruno Patris, Léa Lansade, Benoist Schaal, Alexandra Destrez

**Affiliations:** 1 Development of Olfaction in Cognition and Communication Lab, Centre des Sciences du Goût et de l’Alimentation, CNRS, Université Bourgogne, Institut Agro Dijon, Dijon, France; 2 Unité de Physiologie de la Reproduction et des Comportements, IFCE, Inrae, CNRS, Université de Tours, Nouzilly, France; 3 Institut Agro Dijon, Dijon, France

**Keywords:** Olfaction, Sheep (*Ovis aries*), Interspecific communication, Stress, Human-animal relationships

## Abstract

While sheep can detect and discriminate human emotions through visual and vocal cues, their reaction to human body odors remains unknown. The present study aimed to determine whether sheep (*Ovis aries*) can detect human odors, olfactorily discriminate stressed from non-stressed individuals, and behave accordingly based on the emotional valence of the odors. Axillary secretions from 34 students were collected following an oral examination (stress odor) or a regular class (non-stress odor). Fourteen female and 15 male lambs were then exposed to these odors through a habituation-dishabituation procedure. The habituation stimulus was presented four times for one minute, followed by the dishabituation stimulus presented once for one minute. Behavioral variables included spatiality relative to target odors, approach/withdrawal, ear positioning, sniffing, ingestion, and vocalization. Both female and male lambs more often positioned their ears backwards/forwards, and asymmetrically when exposed to the dishabituation stimulus, but regardless of their stress or non-stress value. They also changed their approach behavior when exposed to the dishabituation stimuli. Lambs displayed some behavioral signs of discrimination between the habituation and dishabituation odors, but regardless of their relation to stress or non-stress of human donors. In sum, this exploratory study suggests that young sheep respond negatively to the odor of unfamiliar humans, without showing any specific emotional contagion related to the stress odor. This exploratory study suggests young ovines can detect human body odor, a further step toward understanding the human-sheep relationship.

**Supplementary Information:**

The online version contains supplementary material available at 10.1007/s10071-024-01895-1.

## Introduction

Olfaction is a major sensory player in the guidance and regulation of mammalian behavior and adaptive cognition. Received through multiple chemoreceptor systems (main olfaction, vomerolfaction, chemesthesis), chemical information engages a range of cognitive processes (e.g., sensory biases, habituation, mere-exposure learning, imprinting, associative learning, categorization, preferences) that are crucial to fine-tune various activities that are essential for survival and individual adaptation (orientation, foraging, feeding, selecting shelter; e.g., Nielsen 2017; Signoret et al. 1997). But odor cues and signals conveyed by odorous body secretions/excretions are most obviously involved in intraspecific communication (e.g., Brown and Macdonald 1985; Müller-Schwarze & Silverstein 1983; Sommerville and Broom 1998).

The sense of smell is also engaged in interactions between species, where it helps in sorting preys from non-preys, predators from nonpredators, in detecting danger in the environment (olfactory landscape of fear). Mammals also emit alert chemosignals toward conspecifics in case of stress or confrontation with predators (Apfelbach et al. 2005). For example, sheep and cattle avoid food in the presence of canid fecal scents, even though they are starving (Arnould and Signoret 1993; Arnould et al., 1998; Pfister et al. 1990).

Through the process of taming and domestication, humans have further imposed themselves to a small set of mammals. During this long process, generations of domestic animals could have acquired an ability to sense nested cues of human identity (individuality, sex, age) and to interpret their keepers’ behavior, moods, and perhaps physiological states. Among the complex multisensory cognitions they may form about humans, animals can rely on olfaction to potentially infer their identity, sex, health state or emotional dispositions. Non-domesticated species do sort humans along discrete olfactory categories (e.g., Bates et al. 2007), and one might a fortiori expect that domesticated species do so as well. In fact, cows, pigs, dogs and cats do perceive human individuality on the sole basis of body odors (e.g., Behnke et al. 2021; Berns et al. 2015; Hepper 1988; Horowitz 2020; Koba and Tanida 1999; Sommerville and Broom 2001; Taylor and Davis 1998). Domestic animals also detect and appraise the odor of humans as a function of the quality of their interactions with them or of their emotional state (e.g., Polla et al. 2018; Tamioso et al. 2017).

Humans do indeed emit volatile compounds in their body secretions/excretions, in which odor profiles correlate with their affective states. So far, studies have mainly focused on fear-, anxiety-, aggression-, happiness-, and other non-stress-inducing contexts, leading to convey odor cues assumed to be either negatively- or positively-valenced to humans. These emotion-differentiated body odors are detectable to unfamiliar conspecifics who react to them measurably in behavior, attitudes and brain responses (e.g., Adolph et al. 2010; Albrecht et al. 2011; Gomes and Semin 2021; Gomes et al. 2023; de Groot & Smeets 2017; de Groot et al. 2012, 2015; Lübke and Pause 2015; Mutic et al. 2016; Pause 2023; Zhou and Chen 2009). As humans’ best friends, canines were probably the first human-imprinted animals to be investigated for their human odor-related cognitions (e.g., Albuquerque et al. 2016; Hepper 1988; Horowitz 2016, 2020; Kalmus 1955; Miklosi 2014; Schoon & de Bruin 1994). More recently, dogs were found to be keen enough to olfactorily differentiate human emotional states from axillary secretions (D’Aniello et al. 2021; Wilson et al. 2022). Such results extend to other domestic species, as human scents of emotions were also assessed in horses (Jardat et al. 2023; Sabiniewicz et al. 2020), cattle (Destrez et al. 2021), cats (d’Ingeo et al. 2023), and laboratory mice (Destrez et al. 2021). Furthermore, emotional contagion seemed to occur in some of these animals: dogs (D’Aniello et al. 2018, 2021) and mice (Destrez et al. 2021) do indeed exhibit more stress-related behaviors when exposed to a human odor collected under conditions of fear or stress.

With companion dogs, sheep are one of the earliest domesticated species (e.g., Zeder 2008). Stemming from a natural history of herbivory, gregariousness and prey species, ovines constitute a contrasted model to study animal-human relationships. Their excessive sensitivity to stress and emotional hyper-reactivity make them well-suited to study animal emotions (e.g., Désiré, 2004; Greiveldinger 2007a; Greiveldinger et al. 2007b). Interestingly for our purpose, their social life is controlled by multisensory exchanges that heavily rely on olfaction (e.g., Agamy et al. 2022; Baldwin and Meese 1977; Gelez and Fabre-Nys 2004; Kendrick 2008; Lindsay 1965; Mora-Medina et al. 2016; Poindron et al. 1993). In addition, being bred, fed (sometimes suckled), cared, sheared and protected from birth to death, ovines must have incorporated humans as a significant part of their social Umwelt, and therefore sheep may sense interacting humans not only through distal modalities (olfaction, vision, audition; e.g., Agamy et al. 2022; Beausoleil et al., 2006; Kendrick et al. 1995; Knolle et al. 2017), but also through the proximal tactile inputs (Chaumont et al. 2021; Sokolowski et al., 2023). To our knowledge, whether human odors are informative to sheep has not been investigated, and it is the purpose of the present study to assess whether sheep can discriminate stressed vs. non-stressed humans.

We define stress roughly as the constellation of psychological and physiological responses of individuals exposed to a challenging experience (Fink 2010). Psychological consequences of stress induce rapid and more or less intense physiological reactions affecting all effectors involved in an individual’s adaptive responses, from the central and autonomous nervous systems to endocrine, cardiovascular and muscular systems, and to excretory and secretory pathways. These latter secretory/excretory responses to emotional events externalize biological substrates (e.g., sweat, tears, breath, urine, feces, etc.) that encode odor cues correlated with given emotional feelings, and can lead to odor-based emotional contagion to conspecifics (Carr et al. 1971; Pérez-Manrique et al., 2022) as well as to other cohabiting species. As mentioned above, multiple odor cues can be elicited through stressful challenges in humans, the most studied being those triggered under fear/anxiety or anger often tested against joy/elation or a non-stress state (considered as “neutral” or “control”) (de Groot & Smeets 2017; Gomes and Semin 2021; Lübke and Pause 2015). Here, we will evaluate whether 6-month-old sheep are able to tell apart the axillary odor of unfamiliar humans exposed to the stress caused by an academic exam and a non-stress condition, and whether they modify their behavior accordingly, thus reflecting interspecific emotional contagion. In line with studies in other domestic animals, we expect that sheep will differentiate both chemostimuli by discriminating their affective valence in a habituation procedure. The habituation-dishabituation paradigm will assess the effects of stress and non-stress human odor on sheep, focusing on the progressive acquisition of unfamiliar stimuli and their discrimination from habituated stimuli. We expect to observe different discriminative reactivity and habituation patterns between the two odors, as well as different emotional state congruence depending on the odor presented.

## Animals, materials and methods

### Ethics statement

The present study was approved by the Dijon Animal Experimentation Ethics Committee (CEEA; 105). It was run in a private farm (Arc-sur-Tille, Burgundy, France) in the context of the farmer’s usual husbandry practices. French farmers are strictly enforced by law to follow rules observant of animal welfare (Rural Code, chapter IV, Articles L214–1 to L214–23). The present experimenters accorded with the farmer with respect to animal handling.

Otherwise, several human donors were required to provide a sample of their axillary secretions after being subjected to various emotional conditions. Before involvement, these odor-donors were informed about the aims and methods of the study, and all signed an informed consent form.

### Animals and housing

**Fig. 1:**
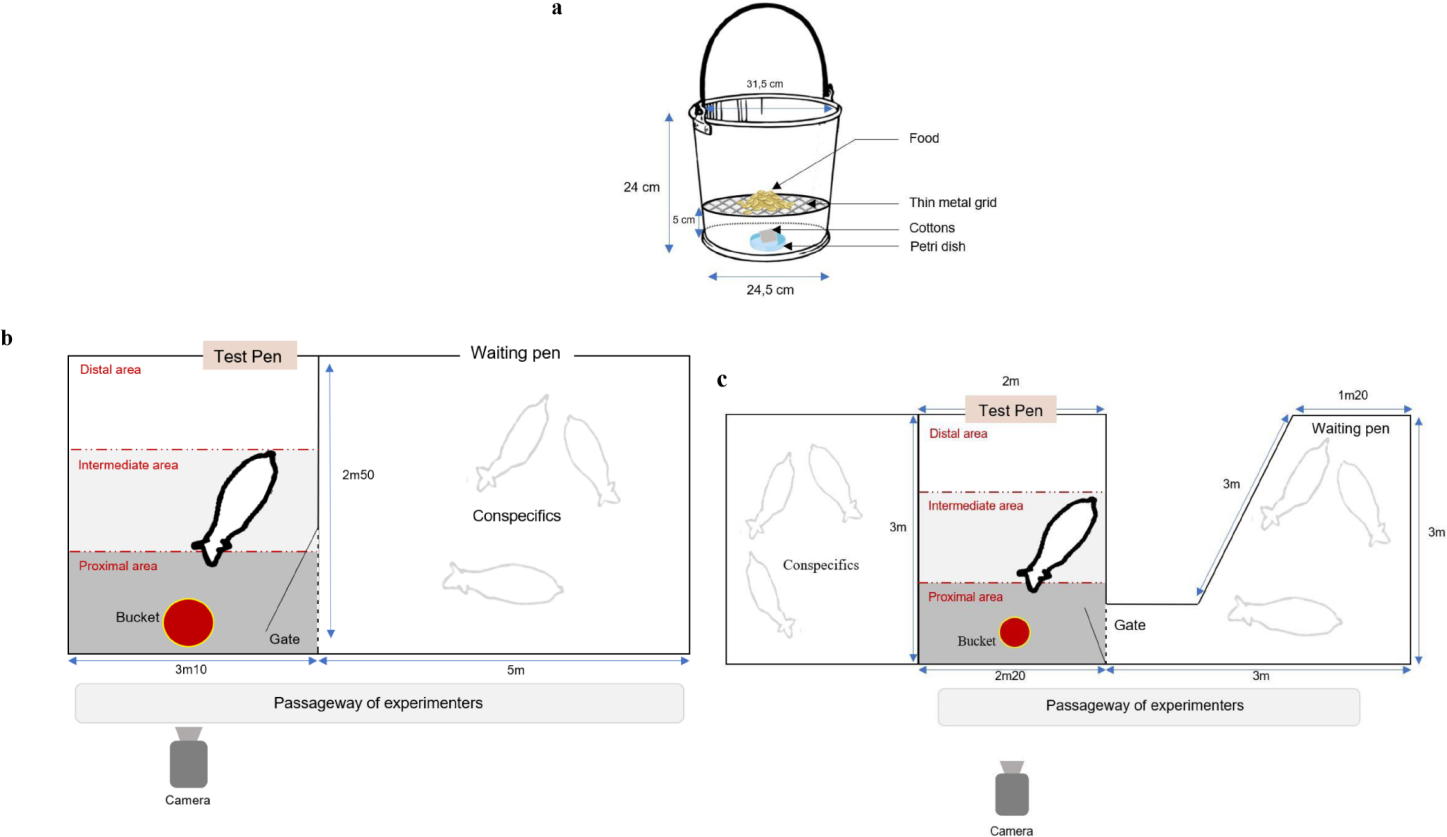
The odor exposure setting for the Habituation-Dishabituation procedure. The odor-dispensing bucket (a); the spatial design of the test pens set up for male (b) and female (c) lambs during the Habituation-Dishabituation test

The study included 70 6-month-old lambs (*Ovis aries*, Ile de France breed; mean age M_age_ = 199, SD_age_ = 9.6 days; 35 females). Males and females were kept separately, females being reared indoors, while the males stayed indoors or outdoors. The test pens were set up in the animals’ familiar enclosures within the breeding barn (indoors). Since both sexes were located in different spaces, slightly different testing arrangements were devised for male and female lambs (see Fig. 1). Fodder was usually distributed in the morning (09:00 am), composed of barley grains, dried alfalfa and *ad libitum* hay, with continuous access to water.

Lambs showing signs of excessive stress (continuous high-pitched bleating, jumping, escape attempts) (*n* = 11) and/or who did not eat in the bucket (*n* = 30) were dropped from the study. After this screening, 29 lambs (14 males, 15 females) were enrolled in the testing phase. They were tested individually, parted from the herd by slated barriers to limit isolation stress, maintaining them in continuous potential contact with peers through vision, audition, touch and olfaction.

### Stimuli

The odor stimuli were sampled from 34 students (31 women, 3 men; M_age_= 22 years, SD_age_ = 3.3 years, range: 20–38 years). Each participant was required to donate axillary odors twice on different days within the same month. One sampling session was done following an important oral examination which was assumed to convey a “stress axillary odor” (SO), the other session following a standard class which was assumed to convey a “non-stress axillary odor” (nSO). Both emotion-inducing contexts occurred in morning time for a duration of two hours (10:00–12:00). The axillae sampling adopted procedures from conceptually similar investigations (e.g., Albrecht et al. 2011; D’Aniello et al. 2018; Destrez et al. 2021; Mujica-Parodi et al. 2009; Sabiniewicz et al. 2020). The day before and during axillary sweat sampling, the donors were instructed not to consume foods known to influence body odor (e.g., garlic, onion, leek, cabbage; e.g., Fialova et al., 2016; Schaal and Porter 1991) or to smoke. None of the participants revealed that they were smokers. They were also required to refrain from using scented hygiene products (deodorant, perfume, scented soap), and to shower only with clear water.

On the day of odor sampling, donors were instructed to wear one of their clean, freshly laundered T-shirts. They were supplied with two cotton pads (8 × 9.5 cm, Tetra Médical, Annonay, France) to be secured under each armpit with strips of scentless tape (Micropore, 3 M, St Paul, USA) during the two hours of emotion-inducing contexts. These cotton pads were then removed by the donors themselves, put in a supplied plastic bag and immediately carried on ice to the lab where they were frozen (-20 °C). The human odor donors self-rated their perceived stress level after the class or oral examination on a 10-point Likert scale as used in other studies on the subject (De Groot et al., 2017; D’Ingeo et al., 2023; Gomes et al. 2020; Jardat et al. 2023; Smeets et al., 2020;). In responding to the question: “What was your stress level during this event?”, the lowest and highest possible self-reported stress level being 1 and 10, respectively. They also filled-in a questionnaire assessing their compliance with the dietary and hygiene instructions. The odor samples were discarded in case of a failure to comply with those instructions or if the difference of the self-perceived stress between the emotionally neutral and negative events was below 2 points. Following these exclusion criteria, 16 female axillary odor donors among 34 were dropped from the study, mainly due to their failure to comply with dietary instructions. So, in the end, 18 participants were retained (15 women, 3 men; M_age_= 22.7, SD_age_ = 1.56 years, range: 20–32 years), reporting an average stress score of 1.66 ± 0.69 in the emotional context assumed to be neutral, and of 7.72 ± 1.27 in the stressful context.

The cotton pads from several donors in the same condition were pooled to attenuate interindividual variability in axillary odors (D’Aniello et al. 2018; de Groot et al. 2015; Gomes et al. 2020; Kamiloğlu et al. 2018; Lanata et al. 2018; Silva et al. 2020). To that aim, each donor’s cotton pads were cut into three 25.3 cm^2^ pieces to be pooled with those of two other individuals of the same sex. Accordingly, 36 axillary odor pools from both axillae were created and stored at -20 °C until testing, which happened within the 4 months following sampling a period shorter than the one during which frozen axillary samples were reported to be stable after sampling (Lenochova et al. 2008). Of these 36 pools, 29 were used for this study, including 6 pools from male participants and 23 from female participants. Given the small number of participants, the menstrual cycle of females was not discriminated.

Before behavioral tests, the axillary odor pools were thawed at room temperature for 30 min in airtight bags. Then, they were put into an odor diffusion device with a food bait (adapted from Arnould and Signoret 1993; Arnould et al., 1998). The pads were placed in a half Petri dish disposed at the bottom of a bucket, underneath a metal grid preventing animals from reaching them (Fig. 1a). As the male and female lambs responded differently to several pilot-tested feedstuffs, they were not exposed to the same bait placed in the bucket (i.e., barley (8 g) for females; fresh alfalfa (4 g) for males). Experimental buckets were dedicated to only one odor stimulus (SO or nSO) to avoid mixing of the target odor stimuli. The axillary pools were changed between each animal, and the diffusion devices were cleaned with water every day.

### Test setting and conditions

The breeder’s usual practices required that we test male and female lambs in different prandial states. Males had to be tested in the afternoon after the morning feed whereas female were tested in the morning before receiving fodder (at 12:00 am).

Three female experimenters operated the testing, one preparing the buckets with the odor stimuli, one gently manipulating the sheep, and one putting the bucket in the test pen and operating the camera. The experimenters manipulating the sheep and the other placing the bucket in the pen and operating the camera were both blind to the odor condition. The experimenters did not smoke before the tests or wear body scents. During testing, all experimenters stood silent at least three meters away from the animals. Between the individual tests, straw impregnated with urine and feces was removed if necessary and the pen re-groomed with fresh straw. The lambs’ behavior in the test pens was recorded using a silent camera (GoPro HERO8 Black) facing the test buckets.

Before the experiment was run, all the lambs were familiarized one time to the test pens, stimulus buckets and experimenters. On the day before the test, they were first conducted by groups of 3 or 4 into the test pen to explore and eat in the bucket (without grid and cotton pads) during 1 min. On the day of the test, they were individually led to the test pen and let free for 1 min to feed in the bucket with the grid, food, and a half Petri dish containing cotton pads without human odor (Fig. 1).

### Behavioral tests

#### The habituation-dishabituation test

The ability of lambs to discriminate between SO and nSO was assessed using a sequential habituation-dishabituation (H-D) test (Aviles-Rosa et al. 2020; Coronas-Samano et al. 2016). In the *habituation phase*, lambs were presented with an odor (habituation stimulus, O1) in four 1-min trials (O1.1, O1.2, O1.3, and O1.4), with 30 s inter-trial intervals. Then, in the *dishabituation* or *test phase*, another odor (dishabituation or test stimulus O2) was presented during 1 min. A drop in behavioral responsiveness was expected during the habituation phase, while a rise of responsiveness was expected to the test stimulus in the dishabituation phase (Aviles-Rosa et al. 2020; Coronas-Samano et al. 2016). H-D testing of individual lambs lasted about 15 min. Odor presentation order was counterbalanced across lambs (Stress-NonStress order: SO in habituation vs. nSO in dishabituation; *n* = 15, 7 females, 7 males; or NonStress-Stress order: nSO in habituation vs. SO in dishabituation; *n* = 14, 8 females, 7 males).

#### Dependent variables

The behavior of the lambs was video-recorded for later frame-by-frame analysis on a computer using the BORIS software (Friard and Gamba 2016). Three independent coders who were blind to the odor conditions analyzed the video-recorded tests.

Since each individual inserted its head and snout into the bucket at least once during each presentation, we considered that each individual was exposed to target odors at each presentation. Several categories of behavioral patterns were coded as defined in earlier studies (see Tables 1 and 2 for definitions), from the most distal and global to the most proximal and detailed reactivity toward the target buckets and the stimuli therein. *First*, the sheep’s attention and attraction to the bucket was coded both in terms of spatial proximity to the bucket (proximal: from 0 to 1 m; intermediate: from 1 to 2 m; distal: from 2 to 3 m), and of investigative actions when in the zone proximal to the bucket (visuo-olfactive exploration, contact with the bucket’s rim, and eating in bucket). *Second*, several behavioral items considered indicative of the animals’ emotional state were analyzed to further evaluate their approach vs. avoidance tendencies to the target buckets as a function of SO or nSO presence. These items involved general patterns of behavior such as whole-body expressiveness (immobility, walking, jumping) and global stress indicators (vocalization, jolt, micturition, defecation). In addition, ear displays were assumed to more finely reflect the lambs’ emotional dispositions toward target stimuli (see Table 2, for detailed definitions). Earlier studies reported that sheep’s negative appraisals are actualized by either backwards or forwards up-positioning of the ear pinnas’ concavity (Boissy et al. 2011), as well as by frequent ear position changes (Reefmann et al. 2009). In contrast, situations inducing rather positive or neutral appraisals were associated with horizontal positioning of both ears, with the pinnas facing the ground (Boissy et al. 2011; Tamioso et al. 2017).

These different behavioral items were coded in terms of occurrence and duration during the five 1-min odor presentation trials. The inter-observer agreement (intra-class correlation) for all variables are provided in Supplemental Table 1, along with descriptive statistics for all variables.

The lambs’ approach/withdrawal tendencies relative to the bucket were characterized in three categories. *First*, a spatial proximity index was devised in 3 different areas explored by individuals in function of the target bucket (Fig. 1), as a change in exploratory pattern may reflect the perception of a stimulus and its habituation to it or not (Wang et al. 2023). *Second*, functionally linked behavioral items were pooled into categories of attraction (sniffing, contact, eating) vs. aversion (arousal, locomotion) relative to the bucket. In this case, the durations and frequencies of items “Sniffing bucket”, “Contact with bucket” and “Eating from bucket” were grouped respectively (separate duration and frequency) into “Attraction behaviors”, while items “Vigilance”, “Jolt” and “Moves” were grouped into “Aversion behaviors”. *Finally*, emotional categories assigned to ear motility were pooled according to their assumed meaning as signs of positive vs. negative responses (Table 2). So, the number of times the ears were positioned “Forward”, “Backward” and “Asymmetric” were aggregated into “Negative affect”, whereas the “Horizontal ears” posture was renamed into “Positive affect”.

If a variable was non-discriminatory, we proceeded to evaluate the subsequent variables, and that, in the order described above.

**Table 1:** Definition of the behavioral items coded during the habituation-dishabituation test, with their assumed emotional categories based on the cited reference

Pattern of actions	Definition	Variables (unit)	Emotional categories	References:
Vocalization	High-pitched bleating with open mouth.	Occurrence	Negative stress, opposition	da Costa et al., 2004; Greiveldinger et al. 2007a Guesdon et al. 2015
Micturition	Visible micturition and associated typical posture	Occurrence	High frequency: anxious state	Monk et al. 2018
Defecation	Visible defecation	Occurrence	High frequency: anxious state	Monk et al. 2018
Jolting	Visible transient contractions of shoulder and/or of posterior occurring with bending of legs or moving legs away from each other.	Occurrence	Fear/negative stress in response to suddenness	Greiveldinger et al. 2007a
Vigilance behavior	Standing still, head erect, ears pointed forward. Starts when the animal immobilizes, ends when it moves again	Occurrence, duration (s)	High proportion: anxiety and fear	Monk et al. 2018
Movement	Quadrupedal movement of whole organism (walking, running, jumping)	Occurrence, duration (s)	Negative stress or exploration	da Costa et al., 2004 Guesdson et al., 2015
Sniffing the bucket	Head and ears facing the bucket, nose between 5 and 10 cm from the bucket	Occurrence, duration (s)	High proportion: interest, preference Low proportion: disinterest, avoidance	
Contact with bucket	Head and ears facing the bucket, nose less than 5 cm from the bucket	Occurrence, duration (s)		
Eating from bucket	Head down in the bucket with obviousjaw motions	Occurrence, duration (s)		

**Table 2:**
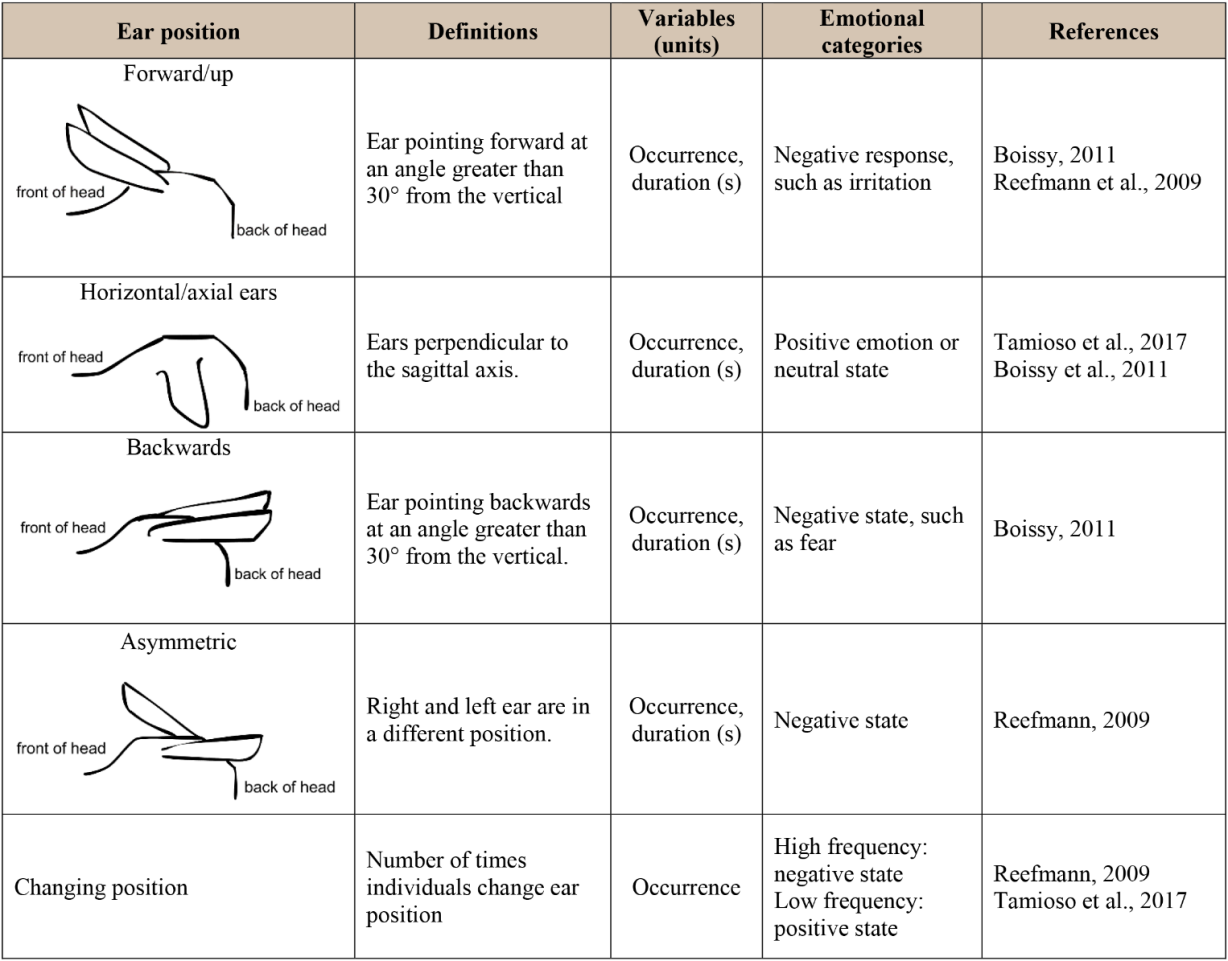
Ear positions in sheep: definitions in relation with emotional states

### Data and statistical analyses

The different prandial condition between the sexes and its potential sensory and motivational correlates (e.g., Aviles-Rosa et al. 2020; Jackson et al. 1999; Verbeek et al., Coronas-Samano et al. 2016), as well as aforementioned contrasted husbandry conditions, prevented us to compare the responses of lambs as a function of sex; hence, separated analyses were run for each sex.

As noted above, the H-D test was composed of two phases: 1/ *habituation*, consisting in presenting four times the same stimulus, from trial O1.1 (first habituation trial) to trial O1.4 (fourth habituation trial), and 2/ *dishabituation*, consisting in trial O2.1. Following Arbuckle et al. (2015) and Yang and Crawley (2009), these two phases were analyzed separately: (i) data from trial O1.1 was compared to those of trial O1.4 to check whether stimulus repetition induced response decrement, actualizing a loss of interest for it; (ii) data from trials O1.4 were compared to those of trial O2.1 to evaluate the level of the animals’ rebound of interest to the novel stimulus, actualizing discrimination between the habituated and novel stimuli.

One statistical outlier on all variables was identified based on Median Absolute Deviation (Leys et al. 2013; i.e. mean score > 3 MAD) and was dropped from subsequent analyses. Therefore, 14 females and 14 males were included in the final statistical analyses of the H-D test. Both occurrences and durations of behavioral data (i.e., locations of the animals in test pen, behaviors and ear positions) were analyzed using a mixed model with restricted maximum likelihood estimation (REML), with animals as random factor and stimulus order in the H-D test (Stress-NonStress or NonStress-Stress), odors (nSO and sO) and sex of the human odor donor as fixed factor for the analyses of the habituation and dishabituation responses (after Bonferroni correction for multiple comparisons). Some variables showed deviations from normality in model residuals (see supplementary material, Supplemental Tables 2 to 5). We nevertheless used linear mixed model to analyze the data as recommended by Knief and Forstmeieir (2021), as it has been demonstrated that (1) linear mixed model are fairly robust to non-normality, (2) deviations from normality usually do not bias regression coefficients, (3) and non-normality of residuals does not impair hypothesis testing (type I error rate is kept as the desired low rate). All analyses were made on Jamovi (α = 0.05, Galluci, 2019; R core team 2022; The Jamovi project 2023).

## Results

### Human participants’ emotional state

As expected, the donors self-reported significantly lower stress levels after attending the normal course than after the oral examination (Wilcoxon test, *α* = 0.05, *n* = 18, *p* < .001), thus validating the stress conditions implemented as inductors of potentially differentiable axillary odor stimuli.

### Habituation-dishabituation test of male lambs

The results are described in Tables 3 and 4, and presented separately for the habituation and dishabituation phases.

*Habituation phase* – Regardless of the habituation odor, the number of times the lambs entered the area proximal to the bucket decreased significantly between trials 1 and 4 (O1.1: 3.15 ± 1.62 times vs. O1.4: 1.71 ± 1.20 times, F_(1,10)_ = 7.91, *p* = .015). They also stayed significantly longer in the intermediate area of the test pen (Table 3). Thus, between habituation trials 1 and 4, male lambs went less into the area containing the odorized bucket (regardless of the odor content) (Table 3). Otherwise, no significant effect of the grade of the odor (nSO or SO) or of the order of odor presentation in the H-D test was noted on any other behavioral categories (attraction or aversion behaviors, ears’ positions: see Table 3). Finally, no significant effect of the sex of human odor donors emerged in any behavioral measurements (all *p*s *> 0.157*).

*Dishabituation phase* – No significant effect of the odor (SO or nSO) nor of the order of odor presentation in the H-D test was found on the lambs’ proximity to the odorized bucket and on their attraction/aversion behaviors (Table 4). However, the lambs displayed more frequent ear positions indicative of negative emotion in response to the dishabituation odor (O2.1) relative to the habituation odor (O1.4) (O1.4: 6.42 ± 2.98 times vs. O2.1: 8.64 ± 4.97 times; F_(1,10)_ = 5.31, *p* = .039; Table 4). Ear position frequency was not affected by the order of odor presentation in the H-D test (*p* = .674). Finally, the sex of the human odor donors turned out to affect the lambs’ proximity to the bucket (Table 4): they spent more time at intermediate distance from it when odorized with men’s axillary odor (27.84 ± 17.11 s) as opposed to women’s axillary odor (14.05 ± 11.89 s; F_(1,10)_ = 5.26, *p* = .031).

**Table 3:** Descriptive (means ± SD) and statistical results of male lambs in the habituation phase of the habituation-dishabituation test

		Odor Presentation		Mixed Model Results			
		O1.1	O1.4	Odor presentation *p*-values	Odor *p*-values	Sex of odor donor *p*-values	Presentation*Odor *p*-values
Animal’s Positions	D_Proximal area	34.06 ± 16.68	24.47 ± 21.75	0.052	0.915	0.261	0.622
	D_Intermediate area	6.55 ± 6.05	15.07 ± 12.89	**0.045** ^*^	0.583	0.946	0.357
	D_Distal area	18.98 ± 13.86	20.14 ± 17.45	0.651	0.813	0.165	0.568
	O_Proximal area	3.15 ± 1.62	1.71 ± 1.20	**0.015** ^*^	0.289	0.353	0.090
	O_Intermediate area	2.76 ± 2.20	2.5 ± 1.99	0.676	0.989	0.683	0.524
	O_Distal area	2.15 ± 1.51	2.07 ± 1.63	0.969	0.969	0.720	0.701
Behaviors	D_Attraction behaviors	22.57 ± 18.16	6.67 ± 9.96	**0.004** ^**^	0.904	0.178	0.917
	D_ Aversion behaviors	22.97 ± 18.39	27.05 ± 30.35	0.553	0.674	0.256	0.390
	O_ Attraction behaviors	2.5 ± 1.65	1.21 ± 1.42	**0.046** ^*^	1	0.157	0.809
	O_ Aversion behaviors	5.35 ± 3.81	5.21 ± 3.06	0.906	0.876	0.913	0.725
Ears	D_ Positve Emotion	27.68 ± 14.46	23.75 ± 16.13	0.519	0.894	0.395	0.764
	D_ Negative Emotion	29.01 ± 15.19	32.13 ± 15.97	0.614	0.993	0.559	0.718
	O_Positive Emotion	3.78 ± 1.87	3.35 ± 1.39	0.511	0.826	0.879	1
	O_Negative Emotion	6.57 ± 3.89	6.42 ± 2.98	0.905	0.577	0.422	0.477
	O_Total Change of position	10.35 ± 4.40	9.78 ± 3.80	0.724	0.732	0.527	0.598

(D: durations; O: number of occurrences; * *p* < .05, ** *p* < .01; O1.1 and O1.4 refer to the 1st and 4th stimuli in the habituation procedure, see text)

**Table 4:** Descriptive (means ± SD) and statistical results of male lambs in the dishabituation phase of the habituation-dishabituation test

		Odor Presentation		Mixed Model Results			
		O1.4	O2.1	Odor presentation *p*-values	Odor *p*-values	Sex of odor donor *p*-values	Odor presentation*Odor *p*-values
Animal’s Positions	D_Proximal area	24.47 ± 21.75	24.85 ± 18.33	0.956	0.413	0.127	0.796
	D_Intermediate area	15.07 ± 12.89	18.89 ± 15.37	0.475	0.108	**0.031** ^*^	0.687
	D_Distal area	20.14 ± 17.45	16.07 ± 15.08	0.347	0.439	0.828	0.913
	O_Proximal area	1.71 ± 1.20	2.14 ± 0.66	0.253	1	0.331	0.606
	O_Intermediate area	2.5 ± 1.99	2.78 ± 1.71	0.606	1	0.914	0.633
	O_Distal area	2.07 ± 1.63	1.85 ± 1.65	0.433	0.433	0.913	0.689
Behaviors	D_Attraction behaviors	6.675 ± 9.96	7.05 ± 11.04	0.892	0.755	0.251	0.593
	D_ Aversion behaviors	27.05 ± 30.35	26.18 ± 15.78	0.919	0.951	0.506	0.983
	O_ Attraction behaviors	1.21 ± 1.42	1.35 ± 1.21	0.728	0.307	0.333	0.642
	O_ Aversion behaviors	5.21 ± 3.06	6.42 ± 2.76	0.245	0.833	0.941	0.702
Ears	D_ Positve Emotion	23.75 ± 16.13	20.36 ± 12.94	0.434	0.243	0.754	0.250
	D_ Negative Emotion	32.13 ± 15.97	35.92 ± 13.18	0.433	0.240	0.717	0.206
	O_Positive Emotion	3.35 ± 1.39	3.14 ± 1.56	0.713	0.713	0.116	0.541
	O_Negative Emotion	6.42 ± 2.98	8.64 ± 4.97	**0.039** ^*^	0.612	0.408	0.561
	O_Total Change of position	9.78 ± 3.80	11.78 ± 5.22	0.144	0.587	0.711	0.700

(D: durations; O: number of occurrences; * *p* < .05; O1.4 and O2.1 refer to the last habituation stimulus and the dishabituation stimulus, see text)

**Fig. 2:**
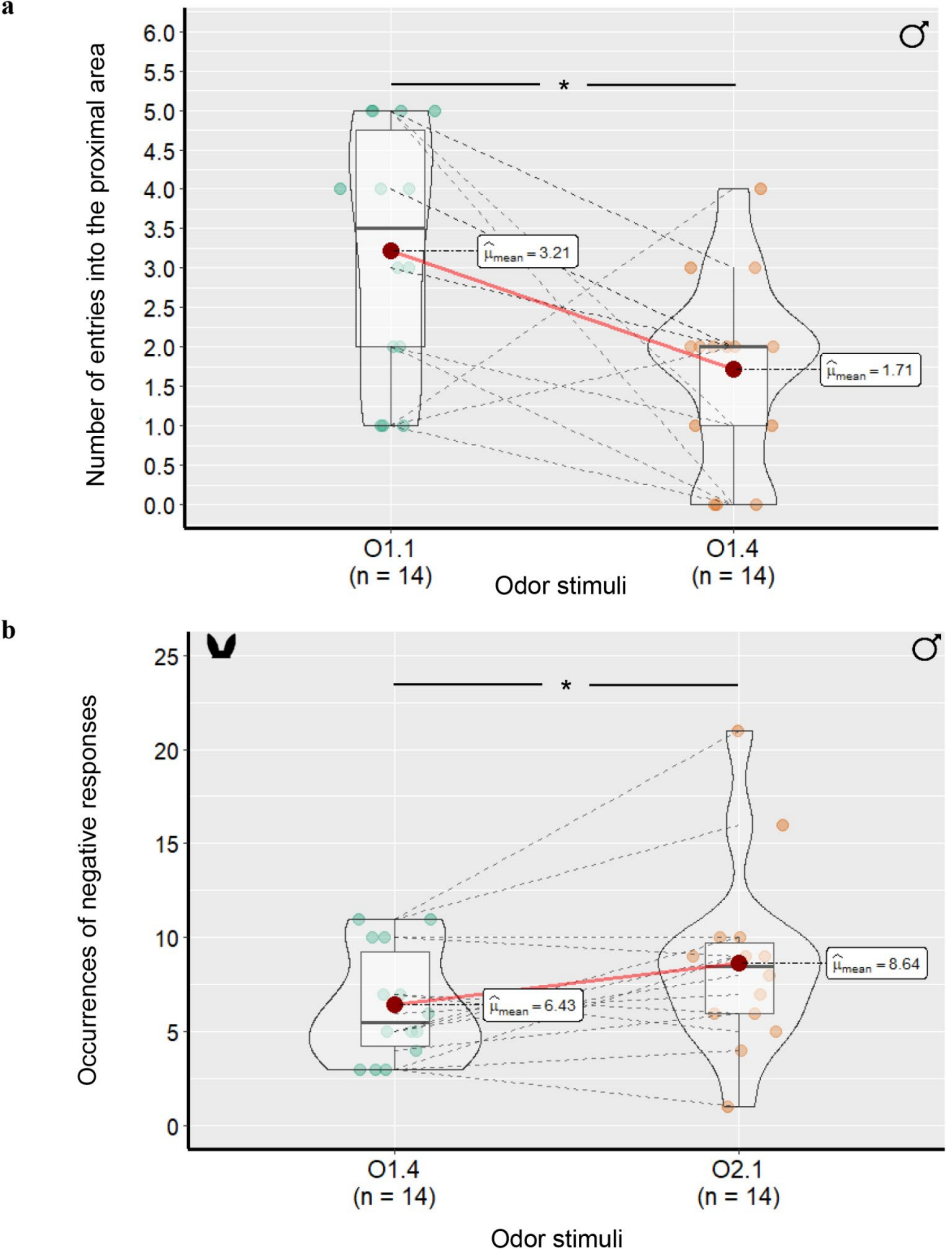
Average number of entries into the proximal area of the bucket during the habituation phase (4 1-min trials) in male lambs (a), and average number of ear-related negative responses in male lambs during the dishabituation phase (b). O1.1 and O1.4: first and fourth presentation of habituation odor, respectively; O1.4 and O2.1: last presentation of the habituation odor and presentation dishabituation odor, respectively (cf. text). Boxplots show the median, first and third quartiles with error bars which represent standard errors. The average is represented by the red bridges, and the other colored dots represent individuals; *: *p* < .05

### Female lambs

The results are described in Tables 5 and 6.

*Habituation phase* – No significant effect of the odor condition (SO or nSO) nor of the odor presentation order were found on any variable (Table 5**)**. Female lambs spent roughly the same amount of time (F_(1,10)_ = 2.09, *p* = .173) in the area proximal to the bucket during habituation trials 1 and 4 (49.30 ± 13.41 s vs. 45.01 ± 11.13 s, respectively), regardless of the odor presented (F_(1,10)_ = 0.44, *p* = .516). The duration of aversion behaviors was affected neither by the odor presentation order (O1.1: 9.46 ± 8.65 s vs. O1.4: 12.52 ± 7.86 s; *p* = .125) nor by the odor condition (O1.1: 9.46 ± 8.65 s vs. O1.4: 12.52 ± 7.86 s; *p* = .246). Finally, no effect of the human odor donors’ sex was found for any of the variables (*p* > .069).

**Fig. 3:**
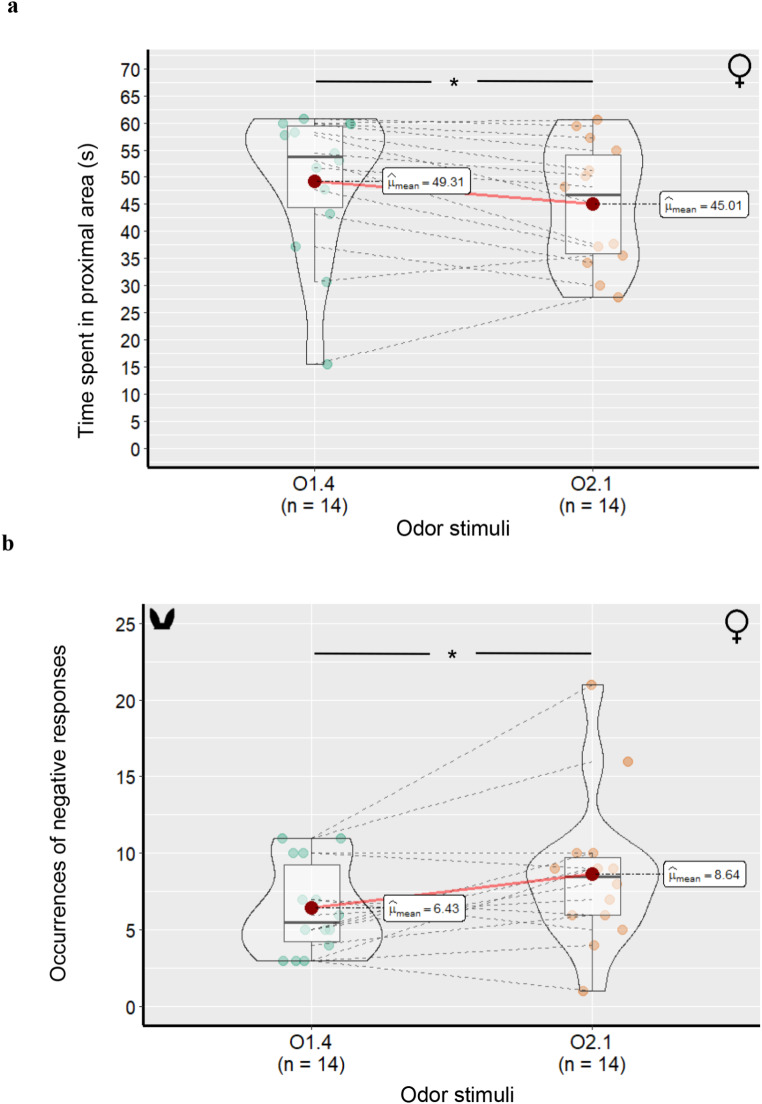
Average time spent by female lambs in the area proximal to the bucket (a), and average number of occurrences of ear-related negative responses during the dishabituation phase (b). O1.4 and O2.1: last presentation of the habituation odor and presentation dishabituation odor, respectively (cf. text). Boxplots show the median, first and third quartiles with error bars which represent standard errors. The average is represented by the red bridges, and the other colored dots represent individuals; *: *p* < .05

*Dishabituation phase* – The lambs spent significantly less time in the area proximal to the bucket when it contained the new odor O2.1 (45.01 ± 11.13 s) compared to the habituation odor O1.4 (49.30 ± 13.41 s, F_(1,10)_ = 5.87; *p* = .032, Fig. 3a). They also entered more often the distal zone when presented with the new odor compared to the habituated odor (2 ± 1.30 vs. 1.28 ± 1.43 times; F_(1,10)_ = 9.13, *p* = .011; Table 6). In addition, lambs more frequently displayed negative emotions as reflected in their ears’ position when the bucket contained the novel odor compared to the habituation odor (O1.4: 6.21 ± 3.16 vs. O2.1: 8 ± 4.77 times, F_(1,10)_ = 4.86, *p* = .047; Fig. 3b). Similarly, they exhibited more change in their ears’ position when presented with the new odor compared to the habituation odor (respectively, 8.92 ± 5.34 times vs. 6.78 ± 3.44 times; F_(1,10)_ = 7.09, *p* = .020). No significant effect of odor condition, nor of the presentation order of the odors during the H-D test was noted on attraction/aversion behaviors or ears’ positions (Table 6). However, female lambs showed longer attraction behaviors when they were exposed to a human male rather than a human female odor stimulus (47.16 ± 10.28 s vs. 28.99 ± 14.82 s, respectively; F_(1,10)_ = 5.60, *p* = .037).

**Table 5:** Descriptive (mean ± SD) and statistical results of the female lambs in the habituation phase of the habituation-dishabituation test

		Odor Presentation		Mixed Model Results			
		O1.1	O1.4	Odor presentation *p*-values	Odor *p*-values	Sex of odor donor *p*-values	Presentation*Odor *p*-values
Animal’s Positions	D_Proximal area	53.15 ± 6.26	49.30 ± 13.41	0.173	0.516	0.272	0.886
	D_Intermediate area	3.05 ± 5.05	3.39 ± 3.21	0.699	0.279	0.719	0.350
	D_Distal area	3.42 ± 3.91	7.23 ± 11.36	0.243	0.719	0.166	0.529
	O_Proximal area	3.85 ± 2.10	4.07 ± 1.43	0.705	0.478	0.801	0.581
	O_Intermediate area	1.5 ± 1.55	2.07 ± 1.81	0.232	0.341	0.379	0.860
	O_Distal area	1.21 ± 0.97	1.28 ± 1.43	0.871	0.556	0.207	0.422
Behaviors	D_Attraction behaviors	39.19 ± 11.38	33.4 ± 14.84	0.182	0.296	0.069	0.410
	D_ Aversion behaviors	9.46 ± 8.65	12.52 ± 7.86	0.125	0.316	0.186	0.330
	O_ Attraction behaviors	4.35 ± 3.97	2.85 ± 1.02	0.311	0.806	0.127	0.538
	O_ Aversion behaviors	3.14 ± 1.29	5.78 ± 3.74	0.133	0.197	0.124	0.424
Ears	D_ Positve Emotion	1.14 ± 1.58	1.06 ± 2.11	0.957	0.349	0.669	0.388
	D_ Negative Emotion	58.06 ± 2.76	55.56 ± 7.25	0.178	0.793	0.602	0.938
	O_Positive Emotion	0.71 ± 0.82	0.57 ± 0.93	0.659	0.670	0.628	0.262
	O_Negative Emotion	5.07 ± 2.97	6.21 ± 3.16	0.207	0.536	0.963	0.981
	O_Total Change of position	5.78 ± 3.21	6.78 ± 3.44	0.265	0.658	0.933	0.749

(D: durations; O: number of occurrences; O1.1 vs. O1.4 refer to the 1st and 4th stimuli in the habituation-dishabituation test; see text)

**Table 6:** Descriptive (mean ± SD) and statistical results of the female lambs in the dishabituation phase of the habituation-dishabituation test

		Odor Presentation		Mixed Model Results			
		O1.4	O2.1	Odor presentation *p*-values	Odor *p*-values	Sex of odor donor *p*-values	Odor presentation*Odor *p*-values
Animal’s Positions	D_Proximal area	49.30 ± 13.41	45.01 ± 11.13	**0.032** ^*^	0.268	0.141	0.808
	D_Intermediate area	3.39 ± 3.21	5.37 ± 6.24	0.152	0.571	0.268	0.539
	D_Distal area	7.23 ± 11.36	9.51 ± 7.57	0.313	0.264	0.136	0.963
	O_Proximal area	4.07 ± 1.43	3.57 ± 2.02	0.210	0.136	0.360	0.315
	O_Intermediate area	2.07 ± 1.81	2.5 ± 1.91	0.072	0.170	0.241	0.731
	O_Distal area	1.28 ± 1.43	2 ± 1.30	0.011*	0.146	0.241	0.887
Behaviors	D_Attraction behaviors	33.4 ± 14.84	32.37 ± 17.15	0.720	0.528	**0.037** ^*^	0.209
	D_ Aversion behaviors	12.52 ± 7.86	16.66 ± 10.17	0.097	0.673	0.081	0.512
	O_ Attraction behaviors	2.85 ± 1.02	2.35 ± 1.21	0.146	0.343	0.142	0.941
	O_ Aversion behaviors	5.78 ± 3.74	7.21 ± 4.87	0.087	0.444	0.077	0.661
Ears	D_ Positve Emotion	1.06 ± 2.11	1.99 ± 3.54	0.448	0.320	0.993	0.859
	D_ Negative Emotion	55.56 ± 7.25	56.41 ± 5.25	0.700	0.936	0.851	0.725
	O_Positive Emotion	0.57 ± 0.93	0.92 ± 1.43	0.401	0.960	0.938	0.428
	O_Negative Emotion	6.21 ± 3.16	8 ± 4.77	**0.047** ^*^	0.758	0.833	0.576
	O_Total Change of position	6.78 ± 3.44	8.92 ± 5.34	**0.020** ^*^	0.737	0.867	0.752

(D: duration; O: number of occurrences; *: *p* < .05; O1.4 and O2.1 refer to the last habituation stimulus and the dishabituation stimulus, see text)

## Discussion

This study examined whether juvenile sheep discriminate the axillary odors of unfamiliar humans subjected to differential induction of emotional states – an assumedly “neutral” situation and a stressful situation self-reported to be clearly negative. The effects of human SO and nSO on sheep were assessed through two processes measurable with the habituation-dishabituation paradigm. First, an animal’s progressive acquisition of an unfamiliar stimulus from initially attention-evoking or fearsome to finally uninteresting or unalarming, and, second, a clear discrimination of a novel stimulus against the habituated stimulus. The possible emotional contagion induced in animals by the information conveyed by the presented odors is also assessed. In that context, our expectations were that, relative to nSO, SO would provoke (i) a differential pattern of habituation in the lambs, and (ii) their discriminative responsiveness between the habituated and the novel stimulus, as well as an (iii) emotional state congruent with the perceived stimulus.

During the habituation phase, male lambs approached less the odorized bucket upon its repeated presentation, decreasing their stay in the area proximal to it and increasing it in the intermediate area. But, considering that the animals were exposed to the odor at each presentation, this growing distance of males to the stimulus bucket, may be interpretable as increasing disinterest or wariness that occurred regardless of the body odor presented in it. Female lambs, on the other hand, stayed close to the odorized bucket, but without effect of its odor content much as their male counterparts. All other behavioral variables in male or female lambs went unaffected by the grade of the odor (SO vs. nSO) provided in the bucket. Thus, no odor-based differential habituation pattern appeared upon the repeated presentation of the odorized bucket, suggesting that the habituation phase of our paradigm was insensitive to reveal the subtle olfactory differentiation of human SO from nSO. Despite the animals had undergone systematic prior familiarization with the experimental setting (including experimenters), this may not suffice to reduce their neophobia or stress level when tested in relative isolation from the herd. Sheep are indeed susceptible of protracted negative stress responsiveness (in terms of behavior, autonomic and endocrine reactivity) to different handling procedures even after intensive familiarization (Hargreaves and Hutson 1990a, b, c), and prone to strong neophobia in diverse contexts (Beck et al. 2021; Burritt and Provenza 1997; Forkman et al. 2007; Garrett et al. 2021; Pedernera et al. 2022). It may be noted that similar non-habituation to repeated body odor of unfamiliar humans was also noted in another domestic ungulate (Jardat et al. 2023). The apparent contrast between male and female lambs regarding proximity to the odorized bucket may be due in part to their different prandial state, sated males being possibly less motivated to get the small food reward than females who were hungry.

In sum, in the present experimental conditions, in staying at distance from the bucket, male lambs may have been more reactive than females to the axillary odor of unfamiliar humans, but without effect of the donors’ stress status. They were thus either insensitive to the odor contrast, if any, between human SO and nSO, or the novelty of the body odors of unfamiliar humans prevailed in eliciting neophobia or aversion actualized at distance from the odorized bucket. A comparable effect was noted in cats who avoided the odor of unfamiliar humans, regardless of the donors’ emotional state (d’Ingeo et al. 2023). Hence, juvenile lambs (and, in the present conditions, males only), like cats, but unlike horses and dogs (e.g., D’Aniello et al. 2018, 2021; Jardat et al. 2023), may react first to human odors along the higher-order information of familiarity or individuality than along the lower-order information of emotional status. Future investigations should thus assess whether sheep can differentiate familiar from non-familiar human beings, and whether they differentiate emotional body odors sampled from familiar persons (i.e., shepherds).

During the dishabituation phase, i.e. when exposed to the novel odor, female lambs spent significantly less time proximal to the bucket as compared to the habituated odor. In addition, at exposure to the novel human odor, both female and male lambs displayed more ears’ positioning typical of ovine negative emotional expression (e.g., Boissy, 2011; Reefmann et al. 2009). Moreover, female lambs changed their ears’ position more frequently when exposed to the novel odor, reflecting their potential sensing of something uncomfortable due to odor unfamiliarity or unpleasantness (Reefmann et al. 2009). These findings suggest that, at least female, juvenile lambs might discriminate stress from non-stress human odors, and specifically that the axillary odor of an unfamiliar human might trigger ovine negative reactivity. These results are consistent with findings in another ungulate (horse) reporting human emotional odors discrimination ability with a similar H-D procedure (Jardat et al. 2023).

In contrast to female lambs, during the dishabituation phase, male lambs nor avoided the area proximal to the odorized bucket, neither did they modify their ear motility. This sex difference may results from the rams’ weaker general reactivity to aversive stimuli, especially those stemming from humans (Vandenheede and Bouissou 1993). Future studies should systemically examine whether the perception of human emotional odor cues varies according to ovine sex.

### Limitations and future directions

Several limitations are to be raised in this study. *First*, due to constraints inherent to husbandry, only small samples of sheep could be tested. In addition, lambs of either sex had to be considered separately as they faced different housing, grouping and feeding practices. In particular, feeding schedules differed by lambs’ sex, leading to uneven prandial states at testing (sated males vs. hungry females), explaining in part response contrasts between male and female lambs. Otherwise, lambs of both sexes were housed in different sectors of the farm so that different test pens had to be designed to minimize displacement stress. Future studies should obviously standardize husbandries and testing conditions across sexes. *Secondly*, testing individuals is a considerable challenge for a highly gregarious species such as sheep (Apple et al. 1993; Guesdon et al. 2015; Kilgour and De Langen 1970). Our lambs may therefore have been in a high level of stress at the beginning of the test procedure, thus potentially limiting the effects of any additional stress caused by human emotional odors and mitigating the demonstration of ovine sensing of human odors. Future studies should increase familiarization time of individual sheep to the test setting in an attempt to further attenuate their hypercautious behavior. *Thirdly*, the lambs were food-baited to the target bucket, a procedure that may have induced feeding motivation at the expense of their sensing of the target odorants inside (a prior study showed indeed that although predator odors reduce intake, these aversive odors do not prevent sheep from eating; Arnould and Signoret 1993). Here, the food bait may have interfered with ovine aversive sensing of a negative odor cue (of a stressed human). Food-specific attractive odorants may indeed mask volatiles conveyed in human body odors (Endevelt-Shapira et al. 2018; Van Nieuwenburg et al. 2019). Thus, a control condition involving only food or a testing solution devoid of feeding motivation should be implemented. This latter option would also allow for a more precise measurement of olfactory exploration (e.g., sniffing) directed to the target odor. However, although it may have reduced the impact of the body odor, the potential masking effect by the food bait would only attenuate the impact of the emotional odors, but is unlikely to produce by itself a systematic difference between odors conditions, and more importantly for our purpose, between habituation and dishabituation odors. *Fourthly*, the emotional intensity of the target human odors may constitute another reason why lambs responded shallowly to our two odor grades (SO vs. nSO). We tested human axillary odors elicited by acute anxiety against those produced under an emotionally neutral condition. Even though odor donors discriminated their stress level between both axillary sampling sessions, the “neutral emotion” samples might be quantitatively but not necessarily qualitatively different, and the emotional intensity of resulting odors may not be of the highest contrast for ovines in the olfactory noise of a sheepfold. Students in higher education report indeed high baseline stress levels in normal courses (e.g., Akhter and Iqbal 2021; Pitt et al. 2018) and our odor samples corresponding to a so-called “neutral course” might nevertheless convey some stress cues – albeit at lesser level than following an oral examination. Thus, one cannot exclude that both SO and nSO may have been sensed as aversive by sheep. Related studies in other species opted for maximally-contrasted emotional contexts (anxiety/fear vs. joy/elation) to induce human axillary odor cues assumed to convey maximally-contrasted stress signaling. Accordingly, following studies in dogs (D’Aniello et al. 2018, 2021), horses (Jardat et al. 2023; Sabiniewicz et al. 2020) and humans (Calvi et al. 2020; De Groot et al. 2015), it would be interesting to explore the ability of sheep to discriminate between human emotional odors sampled under extremely positive vs. extremely negative stress states. *Finally*, the weak effect of the human odor donor’s sex could add a level of complexity to the cognitive processing of human odor by sheep. This effect was noted here for only two response variables (i.e., duration of attraction in females and duration of entries into intermediate area in males). Attributable to the physiological difference in sweat production between men and women (Doty et al. 1978; Wysocki et al. 2009), this response disparity might relate to the intensity of axillary odors rather than their quality. We do not know yet whether this finding reflects lambs’ discrimination of sex-related human body-odor profiles or whether it is attributable to our imbalanced sample of tested animals (six lambs exposed to men’s odor vs. 22 lambs exposed to women’s odor). This could be attributed to the inclusion of only three male donors, resulting in a single donor pool and consequently a lack of variability in the male samples. Nevertheless, rodent studies show that the sex of human odor donors is influential on the stress responsiveness of receiving animals (e.g., Faraji et al. 2022; Georgiou et al. 2022; Sorge et al. 2014). Accordingly, the ovine olfactory differentiation of human gender, of individual variability and of physiological state (menstrual cycle) may be regarded as topics of interest for future studies.

In conclusion, juvenile sheep may discriminate the odor of human axillary secretions produced under contrasting emotional conditions engaging either negative stress (anxiety) or an emotional state assumed as neutral. While the sheep were expected to express specific behaviors (i.e. aversive/sniffing behaviors, typical ear positions) when exposed to stress (vs. non-stress) human odors, under the current conditions and our interpretation of the paradigm, they appeared to respond similarly to the odors of unfamiliar humans, regardless of odor grade. Overall, the odors of unfamiliar humans appeared to convey negative reactions in these young ovines.

To further inform our understanding of the subtle aspects that potentially shape sheep relationships towards humans, future studies are required to unveil their odor-based cognition of humans varying along the interactive dimensions of familiarity/individuality, gender, and emotionality. To that aim, it will be essential to avoid potential interference with competing odors (food) and to implement longer familiarization time to test settings.

## Electronic supplementary material

Below is the link to the electronic supplementary material.

Supplementary Material

## Data Availability

No datasets were generated or analysed during the current study.
