# Supplementary material for: Do sheep (Ovis aries) discriminate human emotional odors?
Source: Anim Cogn. 2024 Jul 26;27(1):51. doi: 10.1007/s10071-024-01895-1 (PMC11282138; doi:10.1007/s10071-024-01895-1)
Supplement: Supplementary file 1 — Supplementary Material [file 10071_2024_1895_MOESM1_ESM.pdf]

Suplementary Materiel

Title: **Do sheep (*Ovis aries*) discriminate human emotional odors?**

Journal Name : Animal Cognition

Izïa Larrigaldie[1], Fabrice Damon[1], Solène Mousqué[1,3], Bruno Patris[1], Léa Lansade[2], Benoist Schaal[1], Alexandra Destrez[1,3]

[1]Dévelopment of Olfaction in Cognition and Communication Lab, Centre des Sciences du Goût, CNRS, Institut Agro Dijon, Univ. Bourgogne, INRAE, Dijon, France

[2] Unité de Physiologie de la Reproduction et des Comportements, CNRS, IFCE, INRAE, Univ. Tours, Nouzilly, France

[3]Institut Agro Dijon, Dijon, France

e-mail address of the corresponding author**: izia.larrigaldie@u-bourgogne.fr**

Supplemental table 1 Intra-class correlation coefficients (ICC2) for the double coding of videos and their 95% confidence intervals (CI)

	**Sexe**	**ICC2 for behaviour frequencies and IC at 95%**	**ICC2 for behaviour durations and IC at 95%**
**Behaviours**	Females	0.96 and [0.95 ; 0.97]	0.98 and [0.97 ; 0.98]
	Males	0.86 and [0.82 ; 0.89]	0.94 and [0.91 ; 0.95]
**Animal's position**	Females	0.71 and [0.59 ; 0.79]	0.97 and [0.96 ; 0.98]
	Males	0.93 and [0.89 ; 0.95]	0.99 and [0.98 ; 0.99]
**Ears positions**	Females	0.78 and [0.68 ; 0.85]	0.95 and [0.93 ; 0.97]
	Males	0.80 and [0.70 ; 0.86]	0.90 and [0.85 ; 0.93]

Supplemental table 2 Results (mean score ± SD) of the habituation-dishabituation test and normality residuals test for male lambs for the habituation phase, with "D" for duration and "O" for occurrence, * p <.05 (O1.1 vs. O1.4 refer to the stimuli in the habituation-dishabituation test, see text)

		Odor Presentation		Mixed Model Results	
		O1.1	O1.4	Shapiro test p-values	Kolmogorov test p-values
Animal' s Positions	D_Proximal area	34.06 ± 16.68	24.47 ± 21.75	.594	.569
	D_Intermediate area	6.55 ± 6.05	15.07 ± 12.89	.131	.856
	D_Distal area	18.98 ± 13.86	20.14 ± 17.45	.602	.837
	O_Proximal area	3.15 ± 1.62	1.71 ± 1.20	.134	.334
	O_Intermediate area	2.76 ± 2.20	2.5 ± 1.99	.879	.953
	O_Distal area	2.15 ± 1.51	2.07 ± 1.63	.259	.759
Behaviors	D_Attraction behaviors	22.57 ± 18.16	6.67 ± 9.96	.092	.433
	D_ Aversion behaviors	22.97 ± 18.39	27.05 ± 30.35	.107	.328
	O_ Attraction behaviors	2.5 ± 1.65	1.21 ± 1.42	**.015***	.617
	O_ Aversion behaviors	5.35 ± 3.81	5.21 ± 3.06	.354	.745
Ears	D_ Positve Emotion	27.68 ± 14.46	23.75 ± 16.13	.298	.95
	D_ Negative Emotion	29.01 ± 15.19	32.13 ± 15.97	.207	.61
	O_Positive Emotion	3.78 ± 1.87	3.35 ± 1.39	.915	1
	O_Negative Emotion	6.57 ± 3.89	6.42 ± 2.98	.088	.981
	O_Total Change of position	10.35 ± 4.40	9.78 ± 3.80	.424	.942

Supplemental table 3 Results (mean score ± SD) of the habituation-dishabituation test and normality residuals test for male lambs for the habituation phase, with "D" for duration and "O" for occurrence, * p <.05 (O1.4 vs. O2.1 refer to the stimuli in the habituation-dishabituation test, see text)

		Odor Presentation		Mixed Model Results	
		O1.4	O2.1	Shapiro test p-values	Kolmogorov test p-values
Animal' s Positions	D_Proximal area	24.47 ± 21.75	24.85 ± 18.33	.577	.995
	D_Intermediate area	15.07 ± 12.89	18.89 ± 15.37	**.034***	.411
	D_Distal area	20.14 ± 17.45	16.07 ± 15.08	.258	.629
	O_Proximal area	1.71 ± 1.20	2.14 ± 0.66	.111	.25
	O_Intermediate area	2.5 ± 1.99	2.78 ± 1.71	.067	.892
	O_Distal area	2.07 ± 1.63	1.85 ± 1.65	.654	.803
Behaviors	D_Attraction behaviors	6.675 ± 9.96	7.05 ± 11.04	**.011***	.176
	D_ Aversion behaviors	27.05 ± 30.35	26.18 ± 15.78	.242	.58
	O_ Attraction behaviors	1.21 ± 1.42	1.35 ± 1.21	**.012***	.617
	O_ Aversion behaviors	5.21 ± 3.06	6.42 ± 2.76	**.024***	.305
Ears	D_ Positve Emotion	23.75 ± 16.13	20.36 ± 12.94	.335	.578
	D_ Negative Emotion	32.13 ± 15.97	35.92 ± 13.18	.534	.971
	O_Positive Emotion	3.35 ± 1.39	3.14 ± 1.56	.664	.691
	O_Negative Emotion	6.42 ± 2.98	8.64 ± 4.97	.369	.905
	O_Total Change of position	9.78 ± 3.80	11.78 ± 5.22	1	.987

Supplemental table 4 Results (mean score ± SD) of the habituation-dishabituation test and normality residuals test for female lambs for the habituation phase, with "D" for duration and "O" for occurrence, * p <.05 (O1.1 vs. O1.4 refer to the stimuli in the habituation-dishabituation test, see text)

		Odor Presentation		Results of residual normality test	
		O1.1	O1.4	Shapiro test p-values	Kolmogorov test p-values
Animal' s Positions	D_Proximal area	53.15 ± 6.26	49.30 ± 13.41	**.004****	.376
	D_Intermediate area	3.05 ± 5.05	3.39 ± 3.21	**<.001*****	.447
	D_Distal area	3.42 ± 3.91	7.23 ± 11.36	**<.001*****	.126
	O_Proximal area	3.85 ± 2.10	4.07 ± 1.43	.141	.71
	O_Intermediate area	1.5 ± 1.55	2.07 ± 1.81	.914	.833
	O_Distal area	1.21 ± 0.97	1.28 ± 1.43	.11	.853
Behaviors	D_Attraction behaviors	39.19 ± 11.38	33.4 ± 14.84	.601	.916
	D_ Aversion behaviors	9.46 ± 8.65	12.52 ± 7.86	.888	.981
	O_ Attraction behaviors	4.35 ± 3.97	2.85 ± 1.02	.822	.986
	O_ Aversion behaviors	3.14 ± 1.29	5.78 ± 3.74	.953	.960
Ears	D_ Positve Emotion	1.14 ± 1.58	1.06 ± 2.11	**<.001*****	.021
	D_ Negative Emotion	58.06 ± 2.76	55.56 ± 7.25	.028	.289
	O_Positive Emotion	0.71 ± 0.82	0.57 ± 0.93	**<.001*****	.087
	O_Negative Emotion	5.07 ± 2.97	6.21 ± 3.16	.403	.87
	O_Total Change of position	5.78 ± 3.21	6.78 ± 3.44	.426	.932

Supplemental table 5 Results (mean score ± SD) of the habituation-dishabituation test and normality residuals test for female lambs for the habituation phase, with "D" for duration and "O" for occurrence, * p <.05, **p <.01, *** p <.001 (O1.4 vs. O2.1 refer to the stimuli in the habituation-dishabituation test, see text)

		Odor Presentation		Results of residual normality test	
		O1.4	O2.1	Shapiro test p-values	Kolmogorov test p-values
Animal's Positions	D_Proximal area	49.30 ± 13.41	45.01 ± 11.13	.15	.702
	D_Intermediate area	3.39 ± 3.21	5.37 ± 6.24	.098	.715
	D_Distal area	7.23 ± 11.36	9.51 ± 7.57	.007	.745
	O_Proximal area	4.07 ± 1.43	3.57 ± 2.02	.926	.995
	O_Intermediate area	2.07 ± 1.81	2.5 ± 1.91	.461	.475
	O_Distal area	1.28 ± 1.43	2 ± 1.30	.857	.966
Behaviors	D_Attraction behaviors	33.4 ± 14.84	32.37 ± 17.15	.326	.617
	D_ Aversion behaviors	12.52 ± 7.86	16.66 ± 10.17	.663	.982
	O_ Attraction behaviors	2.85 ± 1.02	2.35 ± 1.21	.218	.402
	O_ Aversion behaviors	5.78 ± 3.74	7.21 ± 4.87	.621	.938
Ears	D_ Positve Emotion	1.06 ± 2.11	1.99 ± 3.54	**<.001*** **	.131
	D_ Negative Emotion	55.56 ± 7.25	56.41 ± 5.25	**<.001*** **	.447
	O_Positive Emotion	0.57 ± 0.93	0.92 ± 1.43	**.005** **	.334
	O_Negative Emotion	6.21 ± 3.16	8 ± 4.77	.492	.776
	O_Total Change of position	6.78 ± 3.44	8.92 ± 5.34	.567	.85
